# Acoustic and thermal characterization of agar based phantoms used for evaluating focused ultrasound exposures

**DOI:** 10.1186/s40349-017-0093-z

**Published:** 2017-06-01

**Authors:** Georgios Menikou, Christakis Damianou

**Affiliations:** 1 Research Centre for Biomedical Engineering, City, University of London, London, UK; 2 Electrical Engineering Department, Cyprus University of Technology, Limassol, Cyprus

**Keywords:** Ultrasound, Agar, Attenuation, Conductivity

## Abstract

**Background:**

This study describes a series of experimental work completed towards characterizing candidate materials for fabricating brain and muscle tissue mimicking phantoms.

**Methods:**

The acoustic speed, attenuation, impedance, thermal diffusivity, specific heat and thermal conductivity were measured.

**Results:**

The resulting brain (2% w/v agar-1.2% w/v Silica Dioxide-25%v/v evaporated milk) and muscle tissue recipe (2% w/v agar-2% w/v Silica Dioxide-40%v/v evaporated milk) introduced a total attenuation coefficient of 0.59 dB/cm-MHz and 0.99 dB/cm-MHz respectively. Acrylonitrile Butadiene Styrene (ABS) possessed an attenuation coefficient of 16 dB/cm at 1 MHz which was found within the very wide range of attenuation coefficient values of human bones in literature. The thermal conductivity of the brain tissue phantom was estimated at 0.52 W/m°C and at 0.57 W/m.°Cfor the muscle. These values demonstrated that the proposed recipes conducted heat similar to the majority of most soft tissues found from bibliography. The soft tissue phantoms were also evaluated for their thermal repeatability after treating them repeatedly at different locations with the same sonication protocol and configuration. The average coefficient of variation of the maximum temperature at focus between the different locations was 2.6% for the brain phantom and 2.8% for the muscle phantom.

**Conclusions:**

The proposed phantom closely matched the acoustic and thermal properties of tissues. Experiments using MR thermometry demonstrated the usefulness of this phantom to evaluate ultrasonic exposures.

## Background

Phantoms of tissue mimicking materials (TMM) are valuable tools for translating knowledge towards developing ultrasound applications. The effort of researchers in this area is concentrated in the phantom materials used to the mimicked tissue’s acoustic properties. Acoustic properties control the propagation of ultrasound through a medium and therefore a close matching is a prerequisite before translating knowledge reliably to the clinical setting.

A large variety of different TMM is found in literature for replicating soft tissue. Several low cost gelatin gel based recipes have been introduced for mimicking accurately soft tissue for imaging purposes that differ between them in the selection of the additive materials used for controlling acoustic scattering and speed [1–3]. Amongst the reported disadvantages of gelatin gels is their low melting point (35 °C). Unless treated with preservatives gelatin gels are susceptible to microbial and bacterial invasion and difficult to achieve a uniform distribution of the chosen scattering material.

Agar based gels are cheap and easy to produce, durable in high temperatures, nontoxic and disposable but they lack long term stability and repeatability of acoustic properties measurement over time [4–6]. Under ideal storage conditions agar gels can remain stable up to two and a half years [4] and can serve as a perfect replacement for gelatin gels which suffer for temperature dependent structural instability [7].

Polyurethane gels have also been used as alternatives to tissue substitutes. The molecular design of these gels is rather complex and very difficult to standardize. In a reported study a polyurethane TMM phantom was produced with an attenuation coefficient and speed of sound in the lower range of soft tissue values [8].

Polyacrylamide gels have been developed by crosslinking copolymerization of acrylamide and N,N′-methylene bisacrylamide (bis) in an aqueous solution [9]. Polyacrylamide phantoms were doped with bovine serum albumin (BSA) known to enhance acoustic absorption. Attenuation was found to vary linearly with BSA concentration. The gels were optically transparent with adjustable acoustic properties in the lower range of human soft tissues. Fabrication of polyacrylamide gels should be done with caution since it involves handling acrylamide which a known neurotoxin. Acrylamide has been classified as probable carcinogen following evidence from animal studies. A relatively cheaper alternative of BSA doped polyacrylamide gel was described by Takegami et al. [10], where BSA was replaced with egg white. Egg white possesses high protein content and enhances acoustic attenuation via the thermal denaturation process. High egg white concentrations gels induced increased levels of acoustic attenuation and speed compared to the BSA counterparts. Commercially available polyacrylamide gel phantoms manufactured by Onda Corporation are widely used for characterizing transducer geometry, frequency and power profiles. The synthetic gel is transparent and white three dimensional lesions are formed when temperature exceeds 70 °C, making it easy to assess lesion growth, position and shape over time. They are suitable tools for performing quality control tests on HIFU equipment and treatment protocol testing but have fixed acoustic properties and geometry.

N-isopropylacrylamide (NIPAM) gels were designed to become opaque above a threshold temperature, which can be controlled by altering the concentration of acrylic acid (AAC) [11]. By testing different concentrations of AAC, NIPAM based gels were able to match the acoustic and thermal properties of different swine tissues. The NIPAM gels are superior to polyacrylamide BSA or egg white based phantoms since they are reusable. Unlike these phantoms where lesion formation depends on the opaqueness created by protein denaturation which is an irreversible effect, the NIPAM thermal lesions gradually disappear and the phantom gel can be reused.

A polyvinyl alcohol cryogel (PVAC) tissue mimicking phantom gel was presented by Surry et al. [12] suitable for magnetic resonance (MR) and ultrasound (US) imaging. The gel was solidified through a freeze-thaw cycle process and was characterized for the speed of sound which was found in the range from 1520 to 1540 m/s. The gel’s attenuation coefficients were in the range of 0.075–0.28 dB/cm.MHz. T1 and T2 relaxation values were found to be 718–1034 ms and 108–175 ms, respectively. Reinertsen et al. [13] designed a multi-layer phantom made out of PVAC to characterize and correct for brain shift during image guided surgery. Each gel in this study was treated with different number of freeze-thaw cycles to control its mechanical and imaging characteristics.

Magnetic Resonance guided Focused Ultrasound Surgery (MRgFUS) is an emerging therapeutic modality that meets a very wide spectrum of applications in clinical medicine. The purpose of this study is dedicated in describing the methodology of characterizing acoustic and thermal properties of agar and Acrylonitrile Butadiene Styrene (ABS) materials which are used as mimicking phantoms suitable for testing MRgFUS thermal protocols [14, 15]. A novel approach of enhancing acoustic attenuation by doping agar based gels with materials that allow independent control of acoustic absorption and scattering was assessed. The suitability of using ABS as a bone replica was also characterized for its acoustic properties. ABS is a very convenient material to consider since it is used in rapid prototyping of 3D models. The introduction of a bone mimicking phantom in composite phantoms that include also the targeted soft tissue phantom is very important. The constructive and destructive thermal effects that result from the interaction of bone with ultrasound in various MRgFUS applications can be explored under controlled conditions and different configurations. Thermal properties characterization is also crucial before translating results to the clinic since the efficacy of MRgFUS depends on the conversion of acoustic energy to heat and its distribution to the targeted tissue.

## Methods

### Estimation of acoustic properties using immersion techniques

In order to assess the suitability of the materials used to construct tissue mimicking phantoms, a method for assessing their acoustic properties is needed. The most relevant acoustic properties for phantoms designed for MRgFUS is the attenuation coefficient and propagation speed. The first property controls the degree of acoustic energy converted to heat and therefore a close matching with the attenuation coefficient of the replicated tissue is desirable. Acoustic speed along with mass density set the reflection coefficient at every interface and consequently the actual fraction of incident acoustic energy propagating through the phantom is dependent on it. Pulse echo and transmission through methods are broadly used for characterizing the attenuation coefficient and acoustic speed of ultrasound in specimens. They involve submerging the specimen under examination in a tank filled with degassed liquid and all measurements are performed with immersion ultrasonic transducers. The most frequently used modern techniques found from literature are presented below. The techniques differ in experimental configuration, the assumptions taken, the type of signals measured and their advantages.

### Transmission through method for measuring acoustic attenuation coefficient

**Fig. 1:**
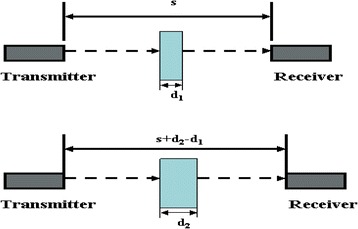
Variable thickness technique for measuring attenuation coefficient of the tested material [9]

This technique compares the transmitted signals through material specimens of different thicknesses [16]. It uses two immersion planar transducers, one for transmitting and one for receiving the signal (Fig. 1). The two transducers operate at the same central frequency and gain to ensure an identical response. The specimen was positioned between the two transducers and preferably beyond the far field of the transmitting transducer, where constructive interference of waves produced at the face of the transducer create a uniform front that decays smoothly with distance. The advantage of the variable thickness method is that there is no need to calculate the reflection or transmission coefficients of the material. First a thinner specimen with thickness ***d***_***1***_ is positioned between the two transducers. The peak-to-peak voltage at the receiver side is measured (***V***_***d1***_) on an oscilloscope. The thicker specimen with thickness ***d***_***2***_ is positioned at exactly the same distance from the transmitter. For strictly correct measurements the receiver in the second measurement must be moved by a ***d***_***2***_ ***-d***_***1***_ distance in order to keep the length of the immersion liquid constant between the two measurements. If water is used as the immersion liquid and the thickness difference between the two specimens is small, we can neglect this correction since this will not attenuate the beam significantly. The peak-to-peak voltage of the thicker specimen is also measured (***V***_***d2***_). The voltage measured at the receiver is directly proportional to the pressure exerted by the acoustic wave. It is then safe to deduce that the square of the voltage measured is proportional to the sound wave’s intensity. If we follow some mathematical analysis to calculate the sound intensity at the receiver’s end for both thicknesses we end up with Eq. 1 for calculating the characteristic attenuation coefficient *(* ***α*** *)* of the material in units of dB/cm.

1 $$ \alpha = \frac{20}{d_2-{d}_1}\left( lo{g}_{10}\frac{V_{d_2}}{V_{d_1}}\right) $$

The expression above contains no reflection or transmission coefficient. The coefficients cancel each other out when we calculate the ratio of intensities as a function of the measured voltages for the two different thicknesses.

### Pulse echo method for measuring acoustic attenuation coefficient

**Fig. 2:**
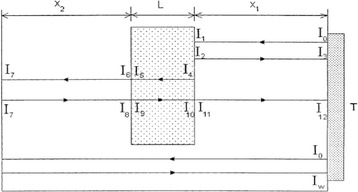
Single transducer pulse echo method [10]

The method introduced by Youssef and Gobran [17], follows a different approach from the variable thickness technique. The main difference is that measurements are performed using reflected echoes at a single transducer (***T***) working in transmit and receive mode (Fig. 2).

The authors described equations of the acoustic wave’s intensity arriving at the transducer face for two different scenarios. First the transducer (***T***) is immersed in tank of fixed dimensions and on the opposite side of the tank a perfect reflector is placed. The reflector can be any flat and smooth material with a high acoustic impedance like aluminum or plastic. The transducer measures the signal intensity (***I***_***w***_) arriving in the absence of a specimen in the path of the beam. The intensity of the signal is dependent only on the attenuation coefficient of the immersion liquid and the distance travelled.

In the second scenario, a specimen of thickness ***L*** is positioned in the pathway of the beam. The transducer records echoes rising from reflections produced by every interface met along the wave’s pathway. The echo returning from the front face of the specimen and travelling a distance ***2x***_***1***_ is denoted as intensity ***I***_***3***_, while the signal with intensity ***I***_***12***_ travels the full pathway of distance ***2(x***_***1***_ ***+ L + x***_***2***_ ***).*** A system of equations describing the reflected echoes intensities at every interface ***(I***_***1***_ ***to I***_***12***_ ***)*** is solved and by using multiple substitutions we end up in a simple system of two equations containing two unknown variables.

***I***_***w***_ and ***I***_***3***_ are directly measured from the transducer whereas the term ***r*** ^***2***^ is equal to the reflection coefficient of the liquid/specimen interface. The term ***α***_***1***_ stands for the attenuation coefficient of the immersion liquid. If degassed water is used as the immersion liquid it can be safely assumed that the attenuation of ultrasound is negligible and therefore the exponent approximates unity, which leads to a very simple expression described by Eq. 2.

2 $$ \frac{I_w}{I_3}=\left(\frac{1}{r^2}\right){e}^{\left[-4{a}_1\left({x}_2+ L\right)\right]\kern0.75em } = \left(\frac{1}{r^2}\right),\ f o r\ {a}_1\approx 0 $$

The second equation of the system after all substitutions is given by Eq. 3, where α_2_ refers to the attenuation coefficient of the specimen.

3 $$ \frac{I_{12}}{I_w} = {\left(1-{r}^2\right)}^4\ {e}^{\left[-4\left({a}_2-{a}_1\right) L\right]} = {\left(1-{r}^2\right)}^4\ {e}^{\left(-4{a}_2 L\right)},\ f o r\ {a}_1\approx 0 $$

Once again *I*_*12*_ and *I*_*w*_ are deduced from the transducer’s voltage signals whereas *r* ^*2*^ is calculated from Eq. 2. The attenuation coefficient ***α***_***2***_ is calculated using Eq. 3. The single transducer method is valid if the reflection and transmission coefficients at both faces of the specimen are equal.

### Estimation of attenuation coefficient variation for agar gels doped with different concentrations of SiO2 using transmission through technique

Agar gels (granulated form, microbiology grade– Merck Millipore, Darmstadt, Germany) of a 2% w/v doped with silica dioxide powder of different concentrations (0%, 1%, 2%, 3% w/v) were prepared. The silica particles size ranged between 0.5 and 10 μm (Silica Dioxide, Sigma-Aldrich, St. Louis, Missouri, United States). The variable thickness technique was used because of its simplicity and because it did not require determination of reflection coefficient. For each silica dioxide concentration a set of two samples of different thicknesses (16 mm and 26 mm) were prepared. The samples were poured in plastic 3D printed plastic containers and were left to solidify overnight. Custom made plastic holders were designed and manufactured to accommodate the specimen containers of different thicknesses (16 and 26 mm) in between the immersion transmitter and receiver. The holder had two cylindrical cavities that could fit tightly the two 10 mm circular 3.5 MHz transducers (Etalon, ESN-410-SCBI) facing each other while the specimen was positioned in between and with side openings perpendicular to the acoustic beam’s pathway emitted by the transmitting transducer. The −3 dB bandwidth of the transducers is 1.8 MHz. The transmitter (T/R) and receiver (R) were connected via coaxial cables to a Panametrics pulser/receiver (Panametrics 500PR, Olympus Corp, Tokyo, Japan).

The signal of the receiving transducer was fed to a 20 MHz oscilloscope (Hameg HM203-7, HAMEG Instruments GmbH, Mainhausen, Germany) to observe voltage over time. The attenuation coefficient in dB/cm was calculated using Eq. 1 for every set of gels. The coefficient for different silica concentrations was normalized to more useful units of dB/cm.MHz by dividing with the frequency (3.5 MHz). This approach resulted to almost linear dependence of attenuation of with frequency (n = 1.08) of the prescribed heterogeneous phantom. A similar approach was used in a study by Nam et al. [18], where attenuation data induced by glass bead scatterers of similar size (10–100 μm) in tissue mimicking phantoms fitted almost linearly in the frequency range between 2.5 and 10 MHz.

### Estimation of attenuation coefficient variation for agar gels doped with different concentrations of evaporated milk using transmission through technique

A recipe described by Madsen et al. was used [4], where evaporated milk was added to agar gels to produce a solid gel of very low scatter tissue mimicking material. Evaporated milk is a dehydrated version of fresh milk, where approximately 50% of water is removed. In the absence of silica dioxide from the gel any drop in the intensity of the acoustic field could be attributed primarily to acoustic absorption by the evaporated milk. Four sets of agar-milk gel samples of different milk concentrations (10%, 20%, 40% and 50% v/v) in a 2% w/v agar based gel were prepared. The evaporated milk used was a product by Friesland Campina, Marousi, Greece (NOUNOU condensed milk). According to the manufacturer’s nutritional datasheet, 100 ml of milk included 4.2 g of fats and 3.5 g of proteins. The agar-milk gel was poured and left to solidify overnight in the custom made plastic sample containers (16 mm and 26 mm). The sample containers were used to determine the attenuation coefficient from each agar-milk concentration sample.

### Linear combination of the tested attenuating agents for creating acoustically realistic soft tissue phantom recipes

The two additives used in the tested agar gels enhanced attenuation primarily by a different loss mechanism. The fine silica dioxide powder was considered a pure scatter agent whereas the low scatter evaporated milk was considered a pure acoustic absorber. Therefore soft tissue mimicking agar based gels can be produced using a linear combinations of silica and evaporated milk concentrations depending on the prescribed scatter induced attenuation coefficient (α_sc_) and absorption induced attenuation coefficient (α_abs_). The total attenuation coefficient (α_t_) of the designed gels can be approximated with the following expression.

4 $$ {\alpha}_t={\alpha}_{sc}+{\alpha}_{abs} $$

Recipes for a brain and a muscle mimicking gel phantom were deduced from results based on agar gel attenuation measurements doped with the two additives in previous sections. The targeted values of α_t_, α_sc_ and α_abs_ for human brain [19, 20] and muscle tissue [21, 22] were extracted from literature.

### Estimation of attenuation coefficient of Acrylonitrile Butadiene Styrene (ABS) samples using transmission through technique

ABS was tested as the candidate material for replicating bone tissue. Two square plates of ABS specimens of different thicknesses, 2.5 mm and 5 mm respectively, were produced (4 × 4 cm) using a rapid prototyping machine (Stratasys, Fortus FDM 400mc, Eden Prairie, Minnesota, USA). A solid interior printing style was used to fill up the model completely with raw material without any air gaps. Attenuation from the plastic was expected to be large and therefore in order to measure adequately a signal, specimens’ thicknesses were kept small. The ABS plates were secured tightly in the rails of a custom made holder approximately midway between the transmitter and receiver. This way it was ensured that the beam leaving the transmitter was incident at 90° to the specimen, and only longitudinal propagation was considered. The setup used was identical to the one used while testing the attenuation of agar gels doped with various additives.

### Estimation of acoustic speed in brain and muscle phantom using pulse echo technique

The pulse echo immersion technique was used for estimating the acoustic speed of the brain and muscle agar gel recipes. The method involved one transducer (Etalon, ESN-410-SCBI) operating at a central frequency of 3.5 MHz. Specimens of each recipe with 2.6 cm thickness were prepared and molded in custom made containers similar to the ones used for the attenuation measurements. The transducer-specimen setup was immersed in a tank filled with degassed water.

Acoustic speed in the specimen was estimated by determining the time difference ***Δt*** between the echoes returning from the interfaces of the specimen. More specifically we observed on the oscilloscope an echo ***E***_***1***_ which corresponded to the reflection from the first interface (water/specimen) and ***E***_***2***_ from the second specimen/water interface. The large first peak corresponded to the reflection at transducer/water interface. The time required (***t***_***1***_) for ***E***_***1***_ to bounce on the first interface while travelling for a distance equal to 2***d***_***1***_ in water is given by Eq. 5, where ***v***_***water***_ represents the propagation speed of sound in water.

5 $$ {t}_1=\frac{2{d}_1}{v_{water}} $$

Following the path of *E*_*2*_ an expression that describes ***t***_***2***_ was produced, which describes the time required for the echo to propagate through a distance of ***2d***_***1***_ in water and ***2d***_***2***_ in the specimen and return back to the transducer (Eq. 6). The expression takes in to consideration that ***v***_***water***_ and **v**_***specimen***_ are different.

6 $$ {t}_2=\frac{2{d}_1}{v_{water}} + \frac{2{d}_2}{v_{specimen}} $$

The time difference ***∆t*** between the two echoes can be found by deducting ***t***_***1***_ from ***t***_***2***_ and end up with an expression which is dependent only on ***d***_***2***_ and ***v***_***specimen***_ (Eq. 7).

7 $$ \varDelta t={t}_2-{t}_1 = \frac{2{d}_2}{v_{specimen}} $$

Solving for ***v***_***specimen***_ in Eq. 8 and assuming that ***d***_***2***_ is known, only ***Δt*** needs to be measured using the oscilloscope.

8 $$ {v}_{specimen}=\frac{2{d}_2}{\varDelta t} $$

Specimens of both gel recipes were prepared and molded inside the 2.6 cm containers used for the attenuation measurements. A thicker sample was selected to increase time difference for the echo to travelling echo and produce a more accurate estimation for the acoustic speed in each sample.

### Estimation of acoustic speed in ABS samples using transmission through technique

Estimation of the acoustic speed in ABS specimens using the pulse echo technique was not possible. The main problem with ABS was that only echoes from the front wall of the specimen were detected (***E***_***1***_) by the transceiver and none from the back wall (***E***_***2***_). The main reason for not detecting the reflection echo was due the disturbance of the propagating wave from the inner layers of the specimen and heavy attenuation. Instead transmission through method was used to determine ABS acoustic velocity by observing the change in the time of flight of the transmission signal (S) detected by the receiver. The acoustic speed of the agar-based phantom was also measured using the transmission technique, just to compare the estimate with the pulse-echo method. The difference in acoustic speed using the two methods was within the measurement error.

In the absence of a specimen the time required (***t***_***1***_) for the sound wave (***S***) travelling from the transmitter (***T***) to the receiver (***R***) positioned at distance (***d***_***1***_ ***)*** is given by Eq. 9, where ***v***_***water***_ corresponds to ultrasound velocity in water.

9 $$ {t}_1 = \frac{d_1}{v_{water}} $$

If a specimen of ABS of thickness ***d***_***2***_ is immersed in between the two transducers the time of flight of the transmission signal (***t***_***2***_) is described by Eq. 10, where ***v***_***specimen***_ corresponds to ultrasound speed in the specimen.

10) $$ {t}_2 = \frac{d_1-{d}_2}{v_{water}} + \frac{d_2}{v_{specimen}}=\frac{d_1}{v_{water}} - \frac{d_2}{v_{water}} + \frac{d_2}{v_{specimen}} $$

By substituting Eq. 9 to Eq. 10 we end with a simple expression for calculating ***v***_***specimen***_, given by Eq. 11.

11 $$ {v}_{specimen} = \frac{d_2}{\left({t}_2 - {t}_1\right) + \frac{d_2}{v_{water}}} = \frac{d_2}{\varDelta t + \frac{d_2}{v_{water}}\ } $$

A specimen of intermediate thickness was used (***d***_***2***_ ***= 1 cm)*** to balance between heavy attenuation of attenuation of the transmitted signal by ABS (approximately −56 dB at 3.5 MHz) and sufficient change in the time of flight.

### Mass density measurements using water volume displacement method

The aforementioned phantom recipes were tested to quantify for their mass density. Samples of 100 ml volume for the gel recipe were prepared. The gels were sliced in approximately equal volumes, with dimensions small enough to fit inside a volumetric tube. Each piece was first weighed with a high precision electronic scale (±0.01 g) and then its volume was deduced by measuring water displacement in a volumetric tube (±1 ml). The greatest source of error recognized in these measurements was the precision of the volumetric tube therefore we used specimens as large as possible to minimize the fractional error in volume measurements. Six specimens from the same batch were taken and the average mass density in g/cm was calculated_._

### Estimation of soft tissue phantoms thermal properties using a noninvasive MR thermometry technique

The traditional methods for measuring thermal conductivity (W/m_._°C) of a material can be categorized in steady state and transient methods. In steady state methods measurements are performed while the temperature of the material under examination does not change over time, whereas in transient techniques measurements are done while heating the material. Both methods require specialized equipment like heat sources and sensors of various geometries or well-engineered experimental setups. The aforementioned methods are partially invasive since they require embedding temperature sensors inside the material under examination affecting its structural integrity.

A completely noninvasive approach (Cheng et al. [23]) estimated thermal conductivity with magnetic resonance temperature imaging (MRTI) and focused ultrasound. The method uses focused ultrasound to induce moderate heating to tissue whilst monitoring temperature with MRTI. The authors showed that tissue conductivity is correlated with the rate of radial expansion of the temperature profile across the focus during cooling period in a plane perpendicular to the propagation of sound. More specifically the following expression was derived demonstrating a linear dependence between the rate of squared radius (***R*** ^***2***^ ***(t)***) of the Gaussian temperature profile over time (***t***) from the initiation of the cooling period with conductivity (***k***_***t***_), mass density (***ρ***_***t***_) and specific heat of the tissue sample (***c***_***t***_).

12 $$ \frac{\partial {R}^2(t)}{\partial t} = 4 D = \frac{4{k}_t}{\rho_t{c}_t} $$

D is the thermal diffusivity coefficient in cm^2^/s. Thermal diffusivity determines the speed at which heat will flow from the source to colder surrounding. It is expressed as the ratio of thermal conductivity divided by the product of specific heat capacity and density.

The method described above was used to quantify the thermal properties of the two soft tissue phantom recipes (brain and muscle). A spherically focused ultrasound transducer (Sonic Concepts, Inc., Bothell, Washington, USA) driven by a 750 W amplifier (JJ&A Instruments, Duvall, WA, USA) was used to sonicate the gel phantoms and raise the temperature at focus. The transducer consisted of a single element piezoelectric crystal of 4 cm diameter and with a focal length of 95 cm.

Five temperature profiles post sonication (12, 24, 36, 48 and 60 s) were collected since late Gaussian peaks were masked by noise and it was too difficult and imprecise to fit a Gaussian function. For each profile the coefficients of the best fitted Gaussian function were found by using the least squares regression criterion between raw and modelled data. The standard deviation or sigma (σ) of each fitted Gaussian was used to calculate the full width at half maximum (FWHM) of the Gaussian temperature profile. The FWHM of a Gaussian function and the associated Gaussian radius R can be calculated by Eq. 13.

13 $$ \mathrm{FWHM} \approx 2.3548\ \upsigma \approx 2\mathrm{R} $$

The mean Gaussian radius for each point in time was calculated by averaging the radii of two orthogonal temperature profiles which were 15 pixels long (approximately 15 mm) and centered over the maximum temperature pixel.

Each of the phantom gels was molded inside a rectangular container with an open top to allow propagation of ultrasound. The transducer and gels were positioned inside a plastic tank filled up with degassed water. Samples were heated using the same sonication protocol (Acoustic Power: 25 W, 60 s of sonication duration). Acoustic power conversion efficiency was calculated using previously acquired radiation force calibration data for this particular transducer. The efficiency was calculated at 50% conversion of electric to acoustic power. The phantoms were targeted in their center in order to maintain uniform temperature gradients in all directions. The targeted depth was not exactly the same but this was not important since according to Cheng et al. [23], conductivity only affected the rate of the radial expansion and not the amplitude of the Gaussian profile.

MR temperature imaging was used to monitor temperature elevation and drop during the exposure. A flexible surface imaging coil (GPFLEX coil by General Electric, Milwaukee, USA) was positioned under the water tank. The setup was positioned at the isocentre of a 1.5 Tesla MRI scanner (General Electric Signa Excite, Milwaukee, USA). Temperature changes were calculated using the proton resonance frequency shift method. The acquisition protocol used for thermometry is presented in 2D SPGR sequence: repetition time (TR): 38.5 ms, echo time (TE): 20 ms, receiver bandwidth (rBW): 15 kHz, Matrix: 128 × 128 pixels, Slice Thickness: 5 mm, Number of excitation (NEX): 1, displayed field of view (DFOV): 25 × 25 cm^2^. The thermometry slice was prescribed in a plane perpendicular to sound propagation and at the level of the focus. Following analysis a single thermal map was produced every 12 s.

The heat capacity of each phantom was estimated by using a weighted sum of the heat capacity of the two main ingredients, which were water and evaporated milk. This was a reasonable assumption since both together consisted for more than 95% of the gel’s mass. From literature it was found that the nominal heat capacities for water is 4.19 J/g.°C and for evaporated milk 3.94 J/g.°C [24].

### Thermal repeatability of the brain and muscle mimicking phantoms treated with focused ultrasound

The thermal repeatability performance of each TMM phantom was assessed with MR thermometry. Each phantom was positioned for a top to bottom sonication inside a water tank filled with degassed water. The phantoms were sonicated using a single element MR compatible HIFU transducer (Sonic Concepts, Bothell, Washington, USA) operating at 1.14 MHz. Five Sonications of 25 W-60 s were done in different locations in each phantom.

### Qualitative evaluation of focused ultrasound heating dependence with target depth

A brain mimicking phantom was molded in a step like shape to create three different propagation depths (shallow, medium, deep). The purpose of this study was to demonstrate the correlation of target depth inside the phantom with the degree of attenuation (low, medium, high). More specifically each level differed by 1 cm (medium to shallow and deep to medium) and by 2 cm the deep to shallow. Small tubes filled with sunflower oil were strapped around the phantom as external references to produce phase correction maps for phase shifts developed by hardware instability between reference and ablation images. Lipids protons resonance frequency is immune to temperature increments and can serve as references to exogenous phase shift sources other than heat. The phantom was immersed in a water tank and the transducer was also immersed in a top to down sonication configuration. Sonication protocols are summarized in Table 1.

**Table 1:** Sonication protocols used for evaluating depth phantom

Target depth	Acoustic Power (W)	Duration (s)	Thermometry Plane
Deep	25	60	Coronal
Deep	25	60	Axial
Medium	25	60	Coronal
Medium	25	60	Axial
Shallow	25	60	Coronal
Shallow	25	60	Axial

### Temperature measurement using a thermocouple

A data acquisition board (6251 DAQ, National Instruments, Texas, USA) was used to measure the temperature. An analogue input of the board was used to capture the temperature. An Omega (M2813-1205, OMEGA Engineering, INC. Stamford, Connecticut, USA) voltage to temperature converter was used to measure temperature using a software written in MatLab (The Mathworks Inc., Natick, MA). A thermocouple (Omega engineering) was placed in the material under test in order to measure temperature elevation at the focal point. The size of the thermocouple was chosen to be 50 μm, so that the interaction with the beam of ultrasound was minimized. The DAQ station sampled temperature measurements every 1 s.

## Results

**Fig. 3:**
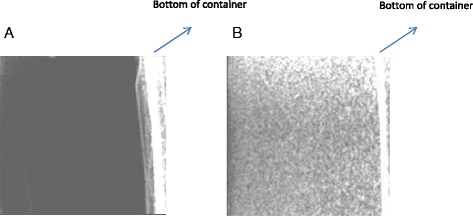
**a** Ultrasound image of pure agarose gel (0% silica) and **b**) Ultrasound image of agarose gel doped with silica (3%)

Two agarose gels (2%w/v) with different silica concentration (0% and 3%) were scanned with a diagnostic ultrasound imaging system (Philips HD7 series Ultrasound Systems, Philips and Neusoft Medical Systems Co. Ltd, Shenyang, China) in order to demonstrate that silica scatters ultrasound. Pure agarose gel (0%) was homogeneously hypoechoic due to the lack of scattering (Fig. 3a). Agarose gel doped with silica (3%) appeared with increased echogenicity due to the significant number of scatters (Fig. 3b).

Using immersion techniques the dependence of the attenuation coefficient for a range of concentrations of silica dioxide and evaporated milk doped in 2% w/v agar gels was examined. The selection of these two materials was done to allow independent scatter and absorption induced attenuation control.

**Fig. 4:**
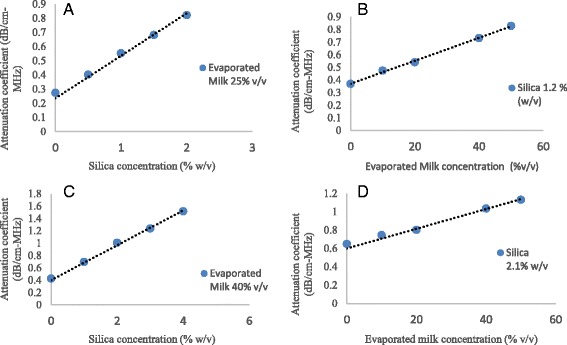
Variation of attenuation coefficient of a 2% w/w agar **a**) 25% v/v evaporated milk gel with different silica concentrations for fabricating the brain phantom. **b** 1.2% w/v silica gel with different evaporated milk concentrations for fabricating the brain phantom, **c**). 40% v/v of evaporated milk with different silica concentrations for fabricating the muscle phantom, **d**) 2.1% w/v silica with different evaporated milk concentrations for fabricating the muscle phantom

Figure 4a shows the variation of attenuation coefficient for 2% w/w agar and 25% v/v evaporated milk gel with different silica concentrations for fabricating the brain phantom. Figure 4b shows attenuation of coefficient for 2% w/w agar with 1.2% w/v silica gel with different evaporated milk concentrations for fabricating the brain phantom. Figure 4c shows the variation of attenuation coefficient for 2% w/w agar and 40% v/v of evaporated milk with different silica concentrations for fabricating the muscle phantom. Finally, fig. 4d shows the variation of attenuation coefficient for 2% w/w agar with 2.1% w/v silica with different evaporated milk concentrations for fabricating the muscle phantom.

Data from attenuation measurements were linearly fitted and the slope was used to calculate the appropriate concentration for each additive depending on the acoustic characteristics of designed tissue mimicking phantom. It was found that the contribution of silica to attenuation (mostly scattering) was 0.28 ± 0.03 dB/cm-MHz per % of silica. The contribution of evaporated milk to attenuation (mostly absorption) was 0.01 dB/cm-MHz per % of evaporated milk. The factors produced from the slopes of the data were used to calculate appropriate recipes for the brain and muscle phantom. It was concluded that based on typical human tissues acoustic properties found from literature the appropriate recipes for the brain and muscle phantoms were Agar 2% w/v-Silica Dioxide 1.2% w/v-Evaporated Milk 25% v/v and Agar 2% w/v-Silica Dioxide 2.1% w/v-Evaporated Milk 40% v/v respectively. The calculated attenuation coefficients of the brain phantom recipe was 0.59 ± 0.05 dB/cm-MHz and 0.99 ± 0.08 dB/cm-MHz for the muscle phantom.

The attenuation coefficient of a common thermoplastic material ABS-M30 (Stratasys Ltd) was investigated to determine its suitability as a bone replicating material for focused ultrasound applications. The same technique was used as for the characterization of agar gels and it was found that the attenuation coefficient was 16.01 ± 6.18 dB/cm-MHz which was well within the very wide range of equivalent values of bone from literature. This large variation in the ABS attenuation measurement was attributed mostly to some variability in the texture of the printed samples that it was impossible to control.

**Fig. 5:**
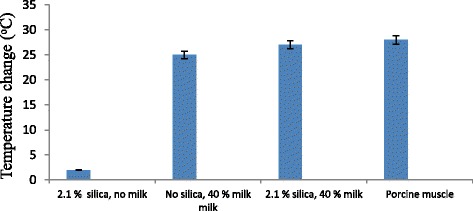
Temperature change versus different phantom recipes and freshly excised porcine muscle, using 20 W for 30 s with the 1.14 MHz spherically focused transducer (focal depth = 2 cm)

Figure 5 shows the temperature change versus different phantom recipes and freshly excised porcine muscle, using 20 W for 30 s with the 1.14 MHz spherically focused transducer (focal depth = 2 cm). The first one includes 2% agar, 2.1% silica (percentage used for muscle) and no evaporation milk. Note that with the presence of silica only, the produced temperature is very low (attributed mostly to agar). The next recipe represents 2% agar, 40% evaporation milk (percentage used for muscle) and no silica. Note that with the presence of sufficient amount of milk, the temperature is increased drastically due to the absorption of milk. The next recipe represents the phantom that mimics muscle (2% agar, 2.1% silica, and 40% evaporation milk). Note that the temperature was slightly increased. Finally, the last data in this graph shows temperature change measured in freshly porcine muscle. Note that the produced temperatures in the muscle phantom and in real muscle are very close.

Acoustic speed and mass density were also assessed for the gel recipes and ABS-M30. Mass density for ABS-M30 was extracted from the manufacturer’s datasheet. Acoustic impedance was calculated as the product of speed with density for all the tested materials. The acoustic properties of the soft tissue mimicking gel phantoms and of the bone mimicking material are summarized in Table 2. The main task was to match the attenuation of the materials of the phantoms (brain, muscle, and bone) to the attenuation reported in the literature. Additionally, we tried to match scattering and absorption as much as possible to the range of values reported in literature which is quite variable.

**Table 2:** Summary of the acoustic properties of soft and bone tissue mimicking materials

	Brain Tissue Mimicking Agar Gel Agar 2% (w/v) + Silica Dioxide 1.2% (w/v) + Evaporated Milk 25% (v/v)	Muscle Tissue Mimicking Agar Gel Agar 2% (w/v) + Silica Dioxide 2.1% (w/v) + Evaporated Milk 40% (v/v)	Bone Mimicking Material ABS-M30 (Stratasys Ltd)
Attenuation Coefficient Scatter enhancing agent (dB/cm-MHz)	0.34 ± 0.04	0.59 ± 0.07	-
Attenuation Coefficient Absorption enhancing agent (dB/cm-MHz)	0.25 ± 0.03	0.40 ± 0.04	-
Attenuation Coefficient Total (dB/cm-MHz)	0.59 ± 0.05	0.99 ± 0.08	16.01 ± 6.18
Acoustic speed (m/s)	1485 ± 12	1529 ± 13	2048 ± 79
Mass Density (g/cm^3^)	1.05 ± 0.01	1.07 ± 0.01	1.04 [38]
Acoustic Impedance(MRayl)	1.56 ± 0.02	1.64 ± 0.02	2.13 ± 0.08

**Fig. 6:**
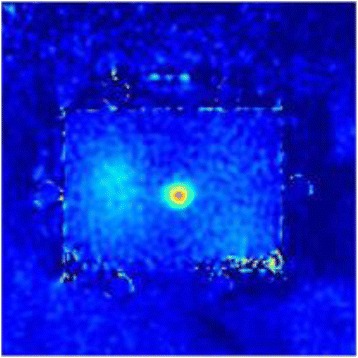
Typical representation 2D thermal map produced in the soft tissue mimicking phantom

Thermal conductivity of each of the gels was assessed by observing the rate of radial expansion of the Gaussian temperature profile at focus. Typical 2D representations of the MRI temperature profile at different times during the cooling period are shown in Fig. 6.

**Fig. 7:**
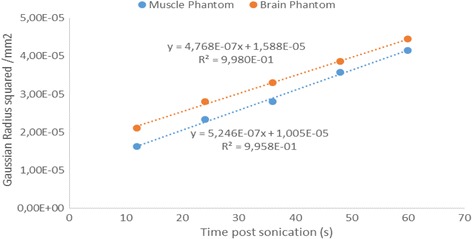
Plot of squared Gaussian radius versus time post sonication

The average squared Gaussian radius of the temperature profile for the brain and muscle tissue mimicking phantom recipes were plotted versus the time post sonication (Fig. 7).

The linear dependence for both sets of data between ***R*** ^***2***^ ***(t)*** and time demonstrated that the radial expansion of the temperature profile “decelerates” with time. The two tested recipes resulted in slightly different slopes as a result of their difference in thermal conductivity. Thermal conductivity was estimated by equating the slopes of the fitted data and the right hand side of Eq. 11. Mass density (*ρ*_*t*_) for each recipe was calculated previously using the water displacement technique. The weighted heat capacity for the brain phantom (75% v/v water-25% v/v evaporated milk) was calculated at 4.13 J/g.°C and for the muscle phantom (60% v/v water - 40% v/v evaporated milk) 4.09 J/g.°C.

By substituting the above to Eq. 11, the thermal conductivities of the brain and muscle phantom were estimated at 0.52 ± 0.06 W/m. °C and 0.57 ± 0.10 W/m. °C respectively. Thermal diffusivity (*D)* which is a thermal property derived by dividing conductivity with density and specific heat capacity, describes how quickly a material reacts to a change in temperature. The calculated diffusivity (*D)* for the brain phantom was 0.0012 ± 0.0001 cm^2^/s and for the muscle phantom 0.0013 ± 0.0001 cm^2^/s. Errors in conductivity and diffusivity coefficients were estimated by considering the error in slope of the fitted data.

**Fig. 8:**
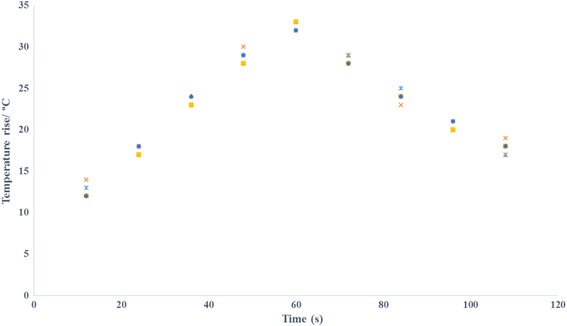
Temperature estimated using MR thermometry vs. time post sonication for the gel phantom mimicking brain

**Fig. 9:**
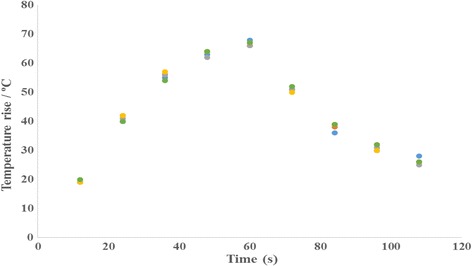
Temperature estimated using MR thermometry versus time post sonication for the gel phantom mimicking muscle

Thermal repeatability of the gels was evaluated by exposing different locations (same depth) of each gel for the same sonication protocol. MR Thermometry was used to record maximum temperature at focus over time and the average coefficient of variation (CV_avg_) for each gel recipe was calculated. Figure 8 shows the temperature estimated using MR thermometry vs. time post sonication for the gel phantom mimicking brain, and Fig. 9 shows the corresponding estimation for the muscle phantom. The CV_avg_ for the brain phantom was calculated at 2.6% and for the muscle phantom at 2.8%.

**Fig. 10:**
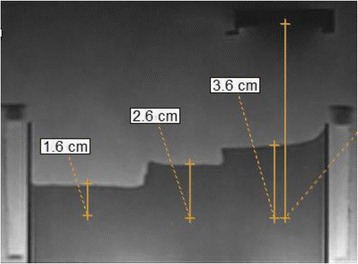
Step like brain phantom for demonstrating the attenuating effect using MR thermometry. Notice the HIFU transducer positioned for a top to bottom sonication

**Fig. 11:**
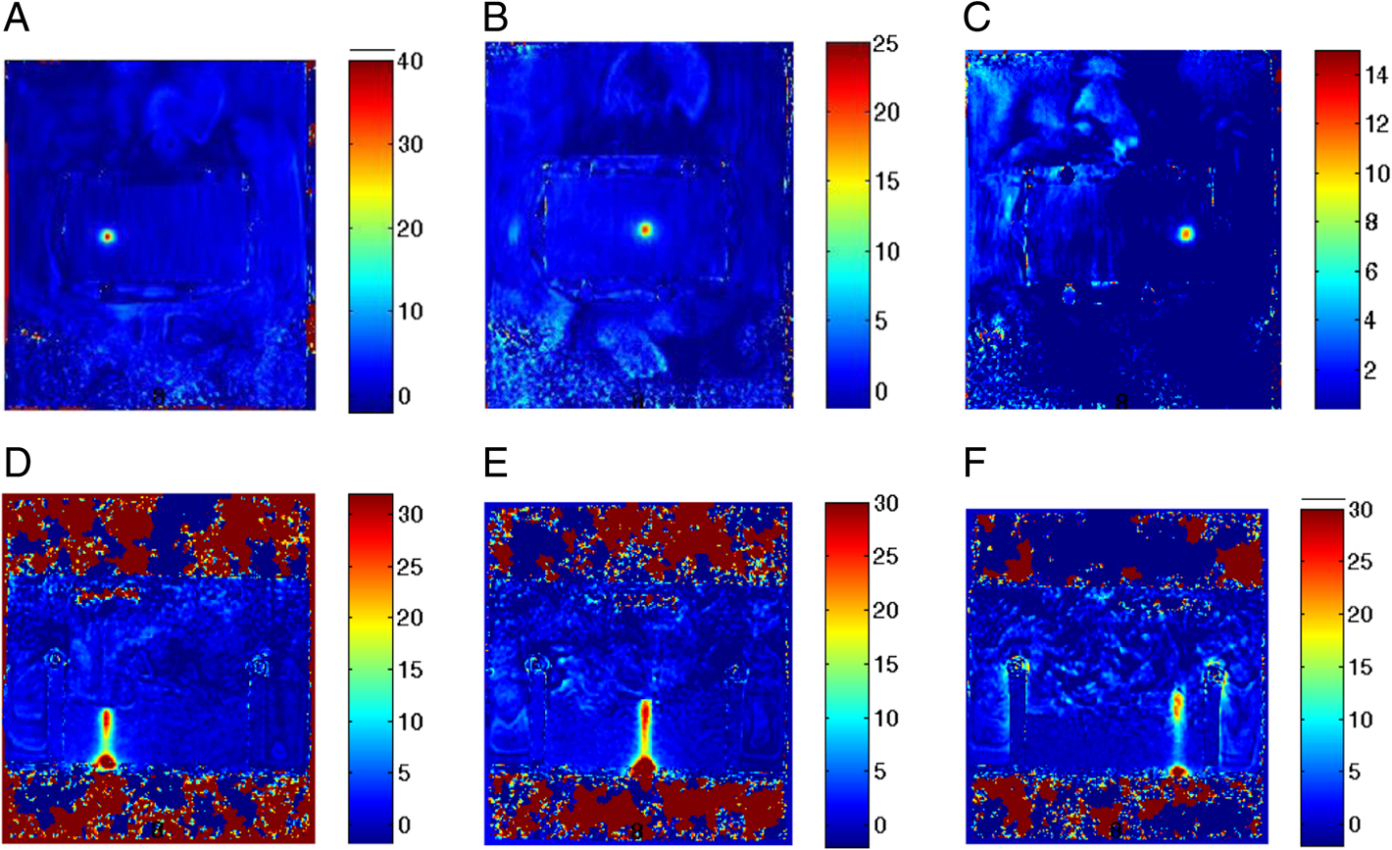
Estimated MR-based temperature in a plane perpendicular to the beam at a depth of **a**) 1.6 cm, **b**) 2.6 cm, and **c**) 3.6 cm respectively. Estimated MR temperature in a plane parallel to the beam at a depth of **d**) 1.6 cm, **e**) 2.6 cm, and **f**). 3.6 cm respectively

The attenuating capability of the brain phantom recipe was demonstrated by introducing different depths of phantom along the propagation pathway in a step like phantom (Fig. 10). MR thermometry was used to calculate the maximum temperature at focus for the same sonication protocol (25 W-60s). Figure 11a shows the estimated MR-based temperature in a plane perpendicular to the beam at a depth of 1.6 cm (maximum temperature was 39 °C). Figure 11b shows the corresponding temperature at a depth of 2.6 cm (maximum temperature was 24 °C), and Fig. 11c shows the corresponding temperature at a depth of 3.6 cm (maximum temperature was 13 °C). Figure 11 d,e,f shows the estimated MR temperature in a plane parallel to the beam at a depth of 1.6 cm, 2.6 cm, and 3.6 cm respectively. It was shown that temperature at focus increased by decreasing phantom depth. This observation proved the attenuating capabilities of the gel phantom. MR thermometry slices parallel to the acoustic beam demonstrated the thermal extend.

## Discussion

This paper described the work done for characterizing important acoustic and thermal properties of materials destined to be used for constructing tissue mimicking phantoms for testing focused ultrasound thermal protocols. The soft tissue replicas were agar gel matrices with additives to control independently from each other, the two main acoustic energy loss mechanisms (scattering and absorption) of sound energy propagating through biological tissue. Agar gels were convenient to work with since they possess a high melting temperature, they are non-toxic and easy to prepare and can be prepared with relatively low cost.

Two calibration curves were calculated by fitting a linear model that characterized attenuation variation with the concentration of either silica dioxide or evaporated milk embedded in agar gels of 2% weight to volume concentration. It was assumed that silica dioxide primarily induced only Rayleigh scattering due to the small size of particles compared to ultrasound’s wavelength. Evaporated milk which was introduced as a very low scattering material, was the primary source of acoustic absorption. For the range of the concentrations tested, the agar-silica dioxide gels demonstrated a linear scattering induced attenuation coefficients of up to 0.85 dB/cm.MHz. Compared with the study of Partanen et al. [4], silica produced a higher degree of attenuation for the same concentration. Similarly agar-evaporated milk gels transmission through assessment resulted in absorption induced attenuation coefficients of up to 0.38 dB/cm.MHz. The slopes of the two calibration curves were used to interpolate and calculate the required concentration for each of the two additive materials to deliver a prescribed total attenuation coefficient while independently controlling the contributions of scattering and absorption.

Based on these calibration curves two soft tissue recipes were prepared. The brain recipe (2% w/w agar, 1.2% w/w SiO_2_, 25% v/v evaporated milk) combined the two additives previously tested to produce a final product with a total attenuation coefficient (0.59 dB/cm.MHz) within the range of bibliographic data [19, 25, 26]. The relative contributions of scattering and absorption of 0.34 dB/cm.MHz and 0.25 dB/cm.MHz respectively were achieved according to attenuation and absorption measurements in mammalian tissues presented by Goss et al. [19] and Sehgal et al. [20]. The muscle phantom recipe (2% w/w agar, 2% w/w SiO_2_, 40% v/v evaporated milk) was designed with an attenuation coefficient equal to 0.99 dB/cm.MHz which agreed with values (0.5–4.1 dB/cm.MHz) found from bibliography [21, 22]. The relatively wide range of attenuation coefficients is due to the fact that muscles are highly anisotropic tissues since they have a strong directional distribution of fibers. Experiments exploring the anisotropy in acoustic speed, attenuation and backscattering in rodents skeletal muscles with fibers orientated at 90° and 45 ° relative to the incident beam were conclusive [27]. It was suggested that the mechanisms which are responsible for anisotropy in these parameters were related to orientation of the elastic tissue structure and to muscle fibers acting as scatterers. It was assumed that there was no directional dependence of attenuation in our muscle phantom recipe since silica particles were orientated randomly inside the gel without any directional prevalence.

ABS was tested as a bone mimicking candidate material and its attenuation coefficient was assessed using the transmission through technique. We have found that ABS was very efficient in attenuating acoustic waves (16 dB/cm/.MHz). The measured attenuation coefficient was within the range of values found in literature. Human bone attenuation data reported in bibliography are very broad as a result of the wide range of porosity, mineralization, bone architecture, choice of anatomical structure and the experimental method followed. The broadband ultrasound attenuation (BUA) coefficient of cortical bone, which forms the compact outer shell surrounding cancellous bone, was measured at different frequencies and ranged between 3.5 and 8.5 dB/cm.MHz [28–33]. In a comprehensive report by Duck et al. [25] skull bone attenuation (all layers) was measured at 22 dB/cm at 1 MHz. Aubry et al. [34] presented a numerical model that correlated skull diploe layer porosity with absorption coefficient which ranged from 2 to 80 dB/cm at 1.5 MHz for the same skull sample.

Measurements of the longitudinal acoustic speed of the two soft tissue recipes were also within the range of biological soft tissues (1478–1595 m/s) [26]. The speed inside the muscle phantom (1529 ± 13 m/s) was significantly higher from the brain’s (1485 ± 12 m/s) because of its higher evaporated milk content. These observations agreed with a recent study by Farrer et al. [35] where acoustic speed in gelatin gels increased with the concentration of evaporated milk concentration. Acoustic speed inside ABS (2048 ± 79 m/s) was in the lower end of a typical range of values found in literature. Culjat et al. [26] reported a speed of 1886 m/s in trabecular bone and of 3476 m/s in cortical bone.

The measured mass density of the muscle phantom (1.07 ± 0.01 g/cm^3^) was found higher from brain (1.05 ± 0.01 g/cm^3^). The densities of both soft tissue recipes were in good agreement of the values reported individually for brain (1.04 g/cm^3^) and muscle (1.09 g/cm^3^) tissue [26]. Density of ABS (1.04 g/cm^3^) was obtained by the manufacturer’s datasheet and was considerably lower from typical cortical bone density (1.97 g/cm^3^).

Acoustic impedance was calculated by multiplying mass density with acoustic speed for each soft tissue recipe and the ABS bone replica. The acoustic impedance of ABS (2.13 ± 0.08 MRayl) was significantly lower from typical cortical bone (7.38 MRayl) [26]. This difference should be taken in to account when using the proposed phantoms for validating numerical models since the degree of reflected energy at the interface will be significantly lower compared to bone.

The thermal properties of the brain and muscle recipes were assessed by correlating the change of the temperature profile’s Gaussian radius over time at a plane perpendicular to the thermal focus formed by a HIFU impulse sonication. The method was described in a publication by Cheng et al. [23]. Thermal conductivity (0.52 ± 0.06 W/m.°C for brain - 0.57 ± 0.10 W/m.°C for muscle) and diffusivity (0.0012 ± 0.0001 cm^2^.s^−1^ for brain - 0.0013 ± 0.0001 cm^2^/s for muscle) for both phantom recipes were found to mimic published values for non-perfused soft tissues. Creeze et al. [36] reported a thermal conductivity range of 0.5–0.6 W/m.°C for non-perfused muscle while in another study by Diederich et al.[37] a range of 0.3–0.7 W/m.°C for unspecified tissue samples was given. Thermal of properties of ABS, which was tested as a bone candidate material, were not assessed since in the majority of focused ultrasound applications energy is delivered to soft tissue. For bone applications apparent temperature is monitored in nearby adjacent soft tissue since bone possesses a very short T_2_ relaxation time and consequently the MR signal produced is short lived.

Evaluation of the brain and muscle phantoms showed that they possess adequate thermal repeatability properties which is essential while investigating hyperthermia applications. The attenuating capabilities of the brain phantom were demonstrated by observing the maximum temperature induced by focused ultrasound at different depths along the acoustic pathway.

## Conclusions

This paper presented full characterization of an agar-based phantom intended for the evaluation of FUS protocols. The evaluation included the measurement of the acoustical, thermal and magnetic properties of the phantom. MR thermometry maps acquired from this phantom showed that the proposed phantom mimics successfully soft tissue. The agar-based material can be used in combination with ABS to mimic different applications such as brain, breast with ribs, bone, and liver with ribs.
